# Using a Digital Parenting Intervention to Prevent Intimate Partner Violence and Promote Gender Equitable Behaviors in South Africa and Jamaica: A Qualitative Study Exploring Partnered Parents’ Experiences of ParentText

**DOI:** 10.1177/10778012251329363

**Published:** 2025-04-13

**Authors:** Moa Schafer, Jamie Lachman, Paula Zinser, Francisco Calderon, Qing Han, Chiara Facciola, Lily Clements, Genevieve Haupt Ronnie, Zimkhita Kame, Zukile Ntentema, Ross Sheil, Jhana Harris, Frances Gardner

**Affiliations:** 1 Centre for Evidence Based Intervention, Department of Social Policy and Intervention, 6396 University of Oxford, Oxford, UK; 2 Social and Public Health Sciences Unit, 3526 University of Glasgow, Glasgow, UK; 3 Centre for Social Science Research, 37716 University of Cape Town, Cape Town, South Africa; 4 Parenting for Lifelong Health, Oxford, UK; 5 IDEMS International, Reading, UK; 6 Social Worker, Cape Town, South Africa; 7 139210 UNICEF Jamaica, Kingston, Jamaica

**Keywords:** intimate partner violence, violence against women, family violence, digital intervention, parenting

## Abstract

This qualitative study explores caregivers’ experiences of using a preprogramed, chatbot parenting intervention with integrated intimate partner violence (IPV) prevention content. The intervention aims to address the challenge of scale-up that in-person parenting programs often face while seeking to reduce both IPV and violence against children simultaneously. Conducted in South Africa and Jamaica, individual interviews and focus group discussions exploring the chatbot IPV prevention content were held with male and female caregivers. The thematic analysis revealed that caregivers perceived the chatbot to be accessible and observed positive changes in relationship dynamics, while also noting some technological barriers and limitations.

Violence against women (VAW) is a public health issue with severe consequences that affect individuals worldwide (World Health Organization (WHO), 2021). Research estimates have revealed that over 27% of women aged over 15 years in intimate partnerships have experienced physical and/or sexual intimate partner violence (IPV) at least once in their lives, underscoring the prevalence of IPV, the most common form of VAW (WHO, 2021). In recent years, there has been an increasing awareness surrounding the co-occurrence of IPV and other forms of violence in the family such as violence against children (VAC) (Pearson et al., 2022). Indeed, IPV and VAC share numerous risk factors, including acceptance of family violence and parental history of abuse, and both forms of violence also have similar short- and long-term consequences (Alhusen et al., 2014; Gracia et al., 2017). Extensive evidence indicates that experiencing IPV is associated with various adverse mental health effects, such as depression, anxiety, and posttraumatic stress disorder (Oram et al., 2022; Prevention Collaborative, 2023; Trevillion et al., 2012). Similarly, research also suggests that experiencing violence in childhood is associated with a range of adverse mental health outcomes, including depression and anxiety (Hillis et al., 2017; Wang et al., 2025). Despite historically being addressed separately, however, following growing awareness of the intersections between IPV and VAC, there have been increasing calls for combined prevention efforts that target both forms of violence simultaneously to achieve a more sustainable impact (Guedes et al., 2016).

Among prevention efforts seeking to address IPV, there is a growing trend toward gender-transformative approaches, which seek to challenge harmful gender norms and promote equitable, nonviolent behaviors and attitudes (Levy et al., 2020). These approaches have shown promise in shifting restrictive gender norms and reducing IPV perpetration (Casey et al., 2018; Doyle et al., 2023; Marcos-Marcos et al., 2023). For instance, a randomized controlled trial (RCT) of the gender-transformative couples intervention “Bandebereho” in Rwanda found that, in comparison to the control group, women in the intervention arm reported less past-year physical IPV, and men and women in the intervention arm also reported less child physical punishment (Doyle et al., 2018). Similarly, an RCT of Stepping Stones and Creating Futures, an intervention combining a gender transformative and economic strengthening approach, found that at a 2-year follow-up, in the intervention group, men's self-reported past year IPV perpetration was lower (Gibbs et al., 2020).

In the field of VAC, parenting programs have emerged as a key strategy for the prevention of family violence, with studies demonstrating promising results in reducing child maltreatment and harsh parenting practices (Backhaus et al., 2023; WHO, 2022). In addition to addressing VAC, parenting programs have also been gaining momentum as an entry point for addressing other forms of violence in the family, such as IPV (UNICEF Innocenti et al., 2023). However, despite robust evidence, scaling up in-person parenting programs poses many challenges, especially in low- and middle-income countries (LMICs) where there are often limited financial resources for development and implementation, restricting the reach of interventions (Sanders et al., 2021). As such, recognizing the potential of using technology and mobile devices to overcome barriers to scale-up, digital parenting interventions have started gaining traction (Awah et al., 2022; David et al., 2024). Indeed, within the field of prevention science, an increasing number of digital and text-messaging-based interventions are being adopted to deliver behavior change interventions (Hall et al., 2015). However, while a rapidly evolving area of research, to date, the existing evidence base on the potential of digital interventions to prevent VAC remains limited (Opie et al., 2023). This is also the case for digitally delivered programs that seek to prevent IPV, which remains a nascent area of research (Anderson et al., 2019). Similarly, the evidence base on digital interventions that seek to prevent *multiple* forms of violence simultaneously is even more scarce, highlighting the need for greater research in this area.

One newly created digital parenting intervention that seeks to prevent multiple forms of violence in the family, including violence against children and IPV, is ParentText, a digital chatbot intervention, that builds on the in-person Parenting for Lifelong Health (PLH) parenting program (Cluver et al., 2018; Ward et al., 2020). The initial format of ParentText did not include IPV prevention content, however, following a new aim to prevent multiple forms of family violence concurrently, content seeking to prevent IPV has been developed and added to the ParentText chatbot, an intervention which was originally designed to focus primarily on reducing violence against children (Schafer et al., 2023). For more information about the structure and content of the ParentText chatbot see Appendix A, and for examples of the IPV prevention content that has been integrated into the intervention (that is, examples of the IPV prevention material that has been added into the preprogrammed messages of the ParentText chatbot), see Appendix B. For further details on how the IPV prevention material was initially developed and added to the structure of ParentText, see Schafer et al. (2023).

The ParentText chatbot with this newly developed IPV prevention content has recently been piloted in a formative, quantitative research study (see Schafer et al., 2025), which was conducted in Jamaica and South Africa, two countries where rates of IPV are particularly concerning. South Africa exhibits one of the highest global rates of IPV, with studies indicating lifetime IPV prevalence ranging from 20% to 50% (Gordon, 2016; Sere et al., 2021; Shai & Sikweyiya, 2015). In Jamaica, rates of violence against women are also alarming, with 24% of women reporting experiences of physical and/or sexual violence from an intimate partner at some stage in their lives (OECD, 2023).

Findings from the formative pilot study in Jamaica and South Africa revealed tentative, yet significant, pre-post reductions in overall IPV experience among women, as well as reductions in men's overall harmful IPV attitudes (Schafer et al., 2025). While the aforementioned pilot study of ParentText focused on IPV outcomes, it did not evaluate the impact on VAC due to limited resources. As such, planned future studies of ParentText aim to also measure the impact on VAC (see Ambrosio et al., 2024). To support additional quantitative research on ParentText, the present qualitative study sets out to gain insights that help to better understand parents’ experiences of using the chatbot, as well as understand how the chatbot and the IPV content are perceived, and explore any potential changes participants observe following the intervention. As such, the present article seeks to answer the overarching research question: How do partnered parents in South Africa and Jamaica perceive their experience interacting with a chatbot parenting intervention? More specifically, it seeks to address the following research questions:

1. What are partnered parents’ experiences of using a chatbot parenting intervention in terms of improving the quality of their partner relationships, reducing conflict, and preventing intimate partner violence?
2. What are the perceptions of partnered parents on the content and structure of ParentText, especially with respect to the ParentText content on relationships?

## Methods

### Study Design

This qualitative study is embedded in a larger research project guided by the Medical Research Council (MRC) framework (Skivington et al., 2021) on the development and testing of the ParentText chatbot intervention, delivered in Jamaica and South Africa (Schafer et al., 2025). The overall aim of the research project, which this qualitative study is nested within, is to examine the feasibility and preliminary effectiveness of the ParentText intervention in the prevention of IPV. As part of the wider research project, a formative, quantitative pre–post pilot of the intervention was conducted, which found reductions in IPV and improvements in attitudes following the program (Schafer et al., 2025). While it was not possible to measure VAC outcomes in the formative evaluation due to resource limitations, effects on VAC outcomes are currently being measured in other trials of ParentText (see for example Ambrosio et al., 2024). The focus of the present qualitative study was to better understand parents’ experiences of using the chatbot, gain insights into how the IPV prevention content was perceived, and explore any potential changes participants observed following the intervention.

In the present study, after participants were enrolled into the chatbot, they received ParentText messages over the course of three weeks, with the main IPV prevention material delivered on Day 2 (see Appendix A and Appendix B). Check-in messages that reminded parents of activities and provided troubleshooting support were also provided (see Appendix C), along with one check-in message per IPV prevention topic, which was sent to users every two days. As a preprogrammed chatbot, the intervention is fully digital, and as discussed further in the limitations section, this means that if users need assistance, even though a troubleshooting support option is available, there are no facilitators available to help users. While this helps keep the chatbot low-cost, it limits options in terms of providing users with support. For more information on the ParentText intervention content see Schafer et al. (2023) and Schafer et al. (2025).

### Study Sites and Participants

The current research was conducted at an urban site in Cape Town in the Western Cape province, in South Africa, and at one urban site and two rural sites across three parishes (Kingston, St. Catherine, St. Elizabeth) in Jamaica. Participants were recruited to take part in ParentText through convenience sampling by local research assistants in Cape Town and by members of UNICEF country office staff in Jamaica. In South Africa, recruitment primarily took place in neighborhoods, townships, and communities in urban Cape Town. In Jamaica, recruitment took place through community and school outreach efforts, and while the aim was to recruit both men and women, due to resource restrictions and challenges reaching men, as described further in the discussion, only women were recruited in Jamaica. Caregivers were eligible for inclusion in the study if they were: over 18 years old in South Africa and over 16 years old in Jamaica (visits to centers for adolescent mothers in Jamaica meant that adolescent parents aged over 16 years old were also included in Jamaica); currently caring for a child between the ages of 0–17 years; being in a relationship (defined as having a partner or being married); having access to a phone that could receive messages on WhatsApp or Telegram and that could connect to the internet or use 3G; provided consent to participate in the study; and able to speak English or isiXhosa (South Africa only).

### The Intervention

ParentText is a chatbot intervention designed for parents and caregivers of children aged 0–17 years, which utilizes automated preprogrammed messaging through platforms such as WhatsApp, Telegram, Facebook Messenger, and SMS for those without smartphones. The parenting content within ParentText is drawn from the in-person Parenting for Lifelong Health parenting programs (Cluver et al., 2018; Ward et al., 2020), which has been adapted together with local partners in various low- and middle-income countries (LMICs), including South Africa, Jamaica, Sri Lanka, Malaysia, and the Philippines to ensure cultural relevance. The present research focuses on the formative evaluation of the ParentText study conducted in Jamaica and South Africa. The adaptation of the PLH material into ParentText and development of the IPV prevention content in South Africa and Jamaica was carried out in collaboration with a range of organizations and research partners, including but not limited to Clowns Without Borders South Africa, Parenting Partners Caribbean, UNICEF Jamaica, and the Jamaica National Parenting Support Commission. Further details on the ParentText adaptation and development process are available in Schafer et al. (2023) and in Ambrosio et al. (2024), and provided in the section below is a brief overview of the qualitative formative study that was conducted, followed by a section with more details on the data collection methods used and the data analysis.

As part of the formative study, participants who were enrolled in the intervention received ParentText messages over three weeks, each tailored to their children's developmental stages. The content was delivered in various formats including text, images, audio, and video, depending on the digital needs of the user, with text-based options to suit phones with less storage space and data, and audio-video content available for users with greater access to data and phone storage. Troubleshooting messages were also provided to participants, encouraging them to practice skills and address any challenges encountered. The parenting content in ParentText focuses on three themes: (1) building positive parent–child relationships through quality time, (2) reinforcing positive child behaviors, and (3) stress reduction skills for parents. Additionally, the chatbot also covers topics such as child development, family finances, and partner relationships (the latter of which seeks to prevent IPV), which is the focus of this study.

The IPV prevention content in ParentText was developed in a separate study (see Schafer et al. (2023)) and is based on addressing key risk factors associated with IPV, and includes the topics: (1) Treat each other as equals; (2) Become a confident parent and supportive spouse; (3) Share family responsibilities; (4) Resolve conflict peacefully; (5) Listen and talk to each other (see Appendix B for an overview of the IPV prevention topics and examples of the messages included). While the ParentText chatbot is aimed toward parents who are in relationships, the present iteration of the chatbot is designed for partners to use individually, rather than together as a couple. As such, while the chatbot addresses partner dynamics, it is not designed as a couples’ intervention (that is, the chatbot is not designed to be used by partners together). Notably, as elaborated in the discussion, this design may have important implications for user engagement and behavior change. The discussion also explores how in future iterations of the chatbot, the intervention might benefit from targeting couples, rather than individuals, to encourage partners to use the chatbot together in order to improve program engagement and effectiveness.

### Data Collection

In total, 20 in-person individual interviews were conducted, with 13 in South Africa (*n* = 8 women; *n* = 5 men) and 7 in Jamaica (*n* = 7 women). In South Africa, four in-person focus group discussions (FGDs) were held, two groups with men (*n* = 7 men) and two groups with women (*n* = 9 women). The interview and FGD guides (see Appendix D) included questions such as “Have you noticed any changes in your relationship with your partner following the program?” and “Was there any specific part or content in the program that you liked or that you found helpful, especially in terms of your relationship with your partner?” While the questions in the individual interviews and the FGDs were not different, the rationale for conducting both individual interviews and FGDs was to gain a greater insight into participants’ experience of using the chatbot. Indeed, the use of qualitative triangulation by combining individual interviews and FGDs has been noted as a key strategy in qualitative research when seeking to gain a more holistic and in-depth insight into understanding a specific experience (Lambert & Loiselle, 2008), which in this case, is users’ experience of the ParentText chatbot. For example, during FGDs, interactions that occur between participants may provide insightful information as part of group discussions that unfold among participants, conversations that may not take place in an individual interview (Cyr, 2015). Simultaneously, research also suggests that individual interviews may help participants feel more comfortable discussing and sharing information on topics that are more sensitive (Kruger et al., 2019). Consequently, using both individual interviews and FGDs allowed for a more thorough exploration of participants’ experience of the chatbot. In South Africa, male interviews and FGDs in South Africa were conducted in English and Xhosa by a male local research assistant. Female interviews and FGDs were conducted in English by the first author with assistance from a local female research assistant interpreter who spoke Xhosa and English. In Jamaica, the female interviews were primarily conducted in English by the first author with interpretation assistance from female and male UNICEF Jamaica staff who spoke English and Jamaican Patois. Interviews and FGDs were audio recorded with consent from participants. Interviews and FGDs in South Africa were anonymized and transcribed and translated into English by a locally hired transcriber who spoke English and Xhosa. Interviews in Jamaica were transcribed and anonymized by the first author.

### Data Analysis

Thematic analysis was conducted following Braun and Clarke's (2006) six-phase process in NVivo 14 (QRS International, 2023). These six steps involve: (1) familiarization with the data; (2) generating initial codes, (3) generating themes; (4) reviewing potential themes; (5) defining and naming themes; and (6) producing the report (Braun and Clarke, 2006). A combined inductive and deductive method was used by drawing upon preexisting theory to interpret the data, while also adopting a data-driven approach throughout the analysis (Braun & Clarke, 2021). As such, while the analysis was data-driven, it is important to acknowledge that the themes that were developed drew upon theoretical assumptions related to the literature on gender equality and violence prevention. Thematic analysis was conducted by the first author, who also discussed the findings with the ParentText research assistants from South Africa and Jamaica, who assisted with data collection and interpretation.

### Trustworthiness and Rigor

Lincoln and Guba's (1985) framework was used to ensure the trustworthiness of the data collected and analyzed. More specifically, the four criteria for assessing trustworthiness proposed by Lincoln and Guba (1985), namely, credibility, transferability, dependability, and confirmability, were used to improve the rigor of the research. To establish credibility, a combination of both individual interviews and FDGs was conducted across the two study contexts as a form of triangulation, and debriefing sessions with the research assistants and interpreters were used to discuss themes to investigate credibility (Rohleder & Lyons, 2017). Transferability was improved by providing a thick description of the participants and the research process, including the setting, sample, demographic characteristics, and inclusion criteria of the study (Korstjens & Moser, 2018). To enhance dependability, an audit trail was maintained throughout the study to record analytical decisions made and to check that data were accurately recorded and stored, with memos written after each data collection and analysis session to keep track of relevant information (Given, 2008). The primary author also kept a reflexive journal with notes to reflect on the influence of their background, interests, and perceptions on the research process (Burke, 2021). Indeed, practicing reflexivity throughout all stages of the research was a critical part of the study. Further reflections and considerations surrounding the primary author's positionality are provided in the positionality section.

### Ethics

Ethical approval was obtained by the Department Research Ethics Committee (DREC) at the University of Oxford (CUREC 2 Ref No: R69569/RE009). Informed consent to participate in the study was obtained from all participants prior to enrollment in the chatbot and before the interviews and FDGs. Confidentiality procedures were also discussed at length with participants prior to the interviews and FDGs, to ensure participants felt safe and comfortable to share their thoughts in front of other participants and the interviewer. Pseudonyms are used to protect confidentiality. Following the interviews and FDGs, participants were automatically provided information on how to access local services that supported individuals experiencing violence. In accordance with UNICEF Risk Communication and Community Engagement Guidelines (UNICEF, 2020), ParentText is also designed to detect high-risk keywords to identify potential disclosure of dangerous situations through the free text field. After detection, ParentText automatically provides participants with an empathetic and empowering reply, with referral details customized to the country, which supports parent and child safety (e.g., hotlines).

### Positionality

The primary author of this study was responsible for designing the study, interviewing most participants, analyzing transcripts, and interpreting the findings. She is a cisgender, Caucasian female, who grew up in southeast Asia, carried out her higher education in Europe, and is currently part of a research institution in the Global North. As such, adopting an open-ended, semistructured interview and focus group design was therefore an important aspect of building rapport with participants. Throughout data analysis, the author actively considered how her own identity as a Caucasian woman, and as someone in a position of social privilege as a researcher part of an academic institution in the Global North impacted her data interpretations in a study involving men and women living in the Global South.

## Results

### Participant Characteristics

In total, 28 participants in relationships (*N* = 16; 53.6% female) took part in the interviews and focus groups (see Table 1). The mean age of participants was 32.5 years. Among women, the majority were married (71.4% in South Africa; 75.0% in Jamaica), and among men, a greater proportion were partnered but not married (83.3%). On average, participants had two children, with a majority working (53.6%) and with a partner who was working (67.9%).

**Table 1.** Participant Demographic Characteristics Across the Two Countries and Overall.

	Demographics expressed as %, count, mean, and SD			
	South Africa		Jamaica	Overall
Demographic characteristic	Female (*n* = 7)	Male (*n* = 12)	Female (*n* = 8)	Total (*n* = 28)
Gender, *n* (%)				
Female	7 (100%)	–	8 (100%)	16 (57.1%)
Male	–	12 (100%)	–	12 (46.4%)
Age, mean (SD)	36 (5.35)	30.1 (3.4)	31.6 (18.2)	32.5 (10.3)
Relationship, *n* (%)				
Married	5 (71.4%)	2 (16.7%)	6 (75.0%)	13 (46.4%)
Partnered but not married	2 (28.6%)	10 (83.3%)	2 (25.0%)	15 (53.6%)
Living with partner (%)	6 (85.7%)	6 (41.7%)	5 (62.5%)	17 (60.7%)
Number of children, mean (SD)	3.43 (0.9)	1.33 (0.8)	1.88 (0.8)	2.07 (1.2)
Education, *n* (%)				
Primary	1 (14.3%)	0 (0%)	4 (50.0%)	5 (17.9%)
Secondary	2 (42.9%)	6 (50.0%)	2 (25.0%)	11 (39.2%)
Higher	4 (28.6%)	6 (50.0%)	2 (25.0%)	9 (32.1%)
Employment, *n* (%)				
Working	4 (57.1%)	8 (66.7%)	3 (37.5%)	15 (53.6%)
Unemployed/looking for work	3 (42.9%)	4 (33.3%)	1 (12.5%)	9 (32.1%)
Student	0 (0%)	0 (0%)	3 (37.5%)	3 (10.7%)
Retired	0 (0%)	0 (0%)	1 (12.5%)	1 (3.6%)
Partner's employment, *n* (%)				
Working	4 (57.1%)	11 (91.7%)	4 (50.0%)	19 (67.9%)
Unemployed/looking for work	3 (42.6%)	1 (33.3%)	2 (25.0%)	7 (25.0%)
Student	0 (0%)	0 (0%)	0 (0%)	0 (0%)
Retired	0 (0%)	0 (0%)	1 (12.5%)	1 (3.6%)
Refuse to answer	0 (0%)	0 (0%)	1 (12.5%)	1 (3.6%)

*Note.* SD: standard deviation

### Themes

The thematic analysis identified three main themes: (1) accessible and nonjudgmental perception of chatbot; (2) changes in relationship dynamics; (3) barriers and design limitations (see Table 2).

**Table 2.** Overview of Themes.

Themes	Sub-theme
Accessible and nonjudgmental perception of chatbot	Flexibility of chatbot and ease of access Anonymity encourages chatbot use
Changes in relationship dynamics	Improved communication and reduced conflict Changes in division of workload Gender role perceptions and changes
Barriers and design limitations	Technological and community barriers Joint chatbot use

#### Accessible and nonjudgemental perception of chatbot

##### Flexibility of chatbot and ease of access

One primary theme that was identified surrounding the chatbot was the convenience of the program and its ease of access:

It was the convenience of the program because I’m more of a person who is always on the phone, so it was convenient for me, even if I’m busy with something, then just, to check the text, just read, just for a full minute, then maybe answer, and get back to what I was doing. It is really convenient (Sifiso, 27-year-old man, South Africa).

Being able to access the chatbot when traveling was also highlighted by participants: “On the road I had access to it” (Alvita, 57-year-old woman, Jamaica). Multiple participants emphasized the ease of access of having the program content delivered via their phone: “Because on your phone it's not a specific time that I have to now dress up, and be in that place at this one time, then, it's anytime in my phone” (Vathiswa, 33-year-old woman, South Africa). In addition, participants expressed that even if they had a support network, the chatbot was convenient because it was accessible in instances when their support network was unavailable or busy: “Sometimes people don't have the time to really sit down and hear what you're going through, cause everybody busy, busy. … It made me feel like somebody cares” (Jayden, 49-year-old woman, Jamaica).

##### Anonymity encourages chatbot use

Many participants, particularly men, shared how they often felt they did not have people around them to turn to for help and that receiving advice and support via the chatbot made them feel like they could receive information without being judged: “Most of us we struggle to talk about our stuff, in like personally to people, because you don’t know what the person is going to say next, so it's better talking to a computer because it won’t judge you, it won’t give you a certain attitude or look” (Zolani, 27-year-old man, South Africa). This sense of comfort was echoed by other participants: “I have nobody that I trust that I usually speak to. So, communicating on my phone was, it was, very comfortable” (Luniko, 32-year-old man, South Africa). The anonymity of the chatbot was also emphasized by participants as something that made them more open to take onboard new advice:

I don’t really have, like, a person I could say that I talk to … The nice thing is that, just, it goes on your phone, and you can’t say who it was … who that just sent you a text, you know, just to motivate you, and … when you’re getting advice from something anonymous, you’re like, ‘Oh, that's a good point.’ (Luniko, 32-year-old man, South Africa)

#### Changes in relationship dynamics

Another theme identified included changes in relationship dynamics which participants experienced after the program.

##### Improved communication and reduced conflict

Participants highlighted improved communication and emotion regulation in times of conflict as a major change after using the chatbot, with some even sharing that the intervention content helped them avoid using violence:

I learned that whenever we are having a conflict, I can leave for a couple of minutes to give my partner time to think about what we are fighting about, and also for me to cool down, because if we continue to argue or fight in the household, nothing will be resolved … I learned to deal with conflict in a positive manner, thus avoiding raising a hand on my partner. (Thembile, 32-year-old man, South Africa)

Others explained how the content on emotion regulation and conflict resolution in the chatbot helped them learn how to communicate better during difficult conversations, and instead of reverting to yelling, how they learned to take a pause and calm down first: “the breathing exercise, that one is good, especially when I've been frustrated … it's helping me take a minute to breathe” (Cedella, 27-year-old woman, Jamaica). Learning to take a pause during conflict was a skill that other participants explained that they had learned from the chatbot too: “It helped us change how we resolved our conflict. We used to yell and shout, however, now if I can see that she is angry, I wait for her to calm down so that we can talk and we manage to resolve our issues in a proper manner, after we’ve calmed down” (Luniko, 32-year-old man, South Africa). This participant also shared how their own behavior changed and approach to conflict and encouraged their partner to change as well:

When she saw that I’m approaching things more differently, then she started following my actions. That's why I said it taught us both. … Once you see that, ‘Oh there's a different approach now’ … in tasks that we usually do, we start sitting down and talking about it, and engaging more with each other than … even with decisions that we usually make. (Luniko, 32-year-old man, South Africa)

This experience was echoed by multiple participants: “He's been changing, he's not getting angry. And I'm not getting angry … Yes. That was good. Before, anything I said to him, we’d get angry and annoyed, frustrated, but now with ParentText, I'm learning a different approach” (Cedella, 27-year-old woman, Jamaica).

##### Changes in division of workload

Participants also shared how they had adjusted their approach to housework and sharing responsibilities following the intervention: “We made a list to help with the house chores, of who should do the dishes, or who will sweep the house. Therefore that has helped us to adjust better and allowed us to resolve the household chores” (Luniko, 32-year-old man, South Africa). This was reiterated by multiple participants, sharing experiences of more equal approaches to housework after using the chatbot: “There's a change, you know. So, while I'm doing the laundry, he’ll be sweeping up outside” (Cedella, 27-year-old woman, Jamaica). Another participant explained how even planning the workload was no longer done by only one person, but rather done together after the program: “After with the program, I managed to share more tips of talking about stuff and then definitely the change has been there … now we started doing things together instead of me telling them what I want or what needed to be done” (Vathiswa, 33-year-old woman, South Africa). Participants also shared perceptions about how the chatbot helped them recognize circumstances where the workload was unequal, which led to more support in the home:

The program helped me, especially with the part where I would do everything alone. This would put pressure on me because I would come back home tired and still try to do everything alone. From cleaning to doing dishes, everything. So this would make me always yell and shout but then with the texts from the program, there was a part where it said, ‘Do you talk to your partner about certain things? Do you share?’ So when I started to do that, everyone started to come up with suggestions. (Bongeka, 40-year-old woman, South Africa)

##### Gender role perceptions and changes

Participants also discussed how they felt their perspectives toward gender roles changed following the digital intervention despite prevailing cultural norms:

There is a cultural myth in our culture that if you help your partner with household chores then they will perceive you in another way. But I’ve learned that no there is no such a thing, and sharing workload with your partner, when she's cooking, you are doing washing. (Ntobeko, 34-year-old man, South Africa)

This shift in perspective toward gender and roles in the home was reiterated by other participants as well: “It removed the mentality that ‘women should stick to the kitchen’, that the ‘department of women’ is the kitchen and cooking and stuff. Me, ‘I can’t do that’, you know, and ‘You, do shopping and stuff’” (Vuyile, 36-year-old man, South Africa). Positive shifts in relationships and gender dynamics were expressed by multiple participants, where improved communication and respect were also perceived to be associated with less conflict: “Communication became stronger and with better communication the respect also started to grow. And I stopped looking down on women. I now look at my partner as my equal. The more respect there is, the more you become happy. With less conflict” (Mandla, 32-year-old man, South Africa). Participants also explained how they started to make more joint decisions after using ParentText: “I can see that the program has changed this. That now I know that when I make a decision, I know that I shouldn’t make that decision alone, I must sit down with her and then we will decide” (Bongani, 28-year-old man, South Africa).

#### Barriers and design limitations

Despite the benefits and positive changes experienced by participants, there were also notable barriers and design limitations, which was the third theme that was identified.

##### Technological and community barriers

To prevent challenges with a lack of storage space and access to data, ParentText was designed with different data and storage options in mind (Schafer et al., 2023). More specifically, participants were able to choose whether to use a text-only or multi-media (i.e., text accompanied by audio, illustrations, or video) version of the intervention. Despite these options, various participants expressed that they faced technological barriers, including challenges with accessing data and limited phone storage: “That was a problem for me, the data, and storing on my phone” (Cedella, 27-year-old woman, Jamaica). This was echoed by others as well, who tried to delete other content on their phone to free up more space: “It fills up my phone. Yeah, that's why. So sometimes I'm like delete, delete, delete” (Martisha, 53-year-old woman, Jamaica).

Participants also shared other environmental problems they experienced, such as challenges charging their phones due to lack of electricity during periodic national blackouts: “I would also experience a problem with the electricity while I’m busy with the programme, then the battery would be low. In our streets the electricity can usually go off for up to like four days” (Amahle, 43-year-old woman, South Africa). In light of these barriers, and other limitations, such as limited phone use during work hours, participants expressed a desire to be able to schedule the timing of messages: “The reason I was behind in the program is that we are not allowed to answer our phones in the workplace” (Zintle, 45-year-old woman, South Africa). The request to schedule messages was also highlighted by others: “It’d be lovely to choose the time” (Vathiswa, 33-year-old woman, South Africa), which was reiterated by others: ‘it’d be better if … it sent messages after six because … if I’m busy I’m not able to pay attention to my phone. But if you do it after six, then I’ll be free and able to’ (Mandla, 32-year-old man, South Africa).

In addition to technological barriers, there were also cultural barriers that influenced the extent to which the chatbot was able to create positive behavior change. For instance, a participant shared an example demonstrating how despite seeing some positive behavioral changes and progress within the home, there were still instances where harmful gender roles and attitudes persisted: “Yes, he cooks now, even though sometimes when I remind him that today it's his turn to cook, he’ll be complaining and reminding me that I am a wife and it's my duty to cook in the household” (Zintle, 45-year-old woman, South Africa). Other participants shared examples where they experienced positive changes in their relationship, but where, harmful gender roles and attitudes returned in contexts outside of the immediate family:

But now the other day, there was his sister in the house, normally … he would change nappies but when the sister is there, it is like, ‘No, you do this.’ Yes, I was very angry. I couldn't even, I couldn’t talk about it, but later on when we were talking, he is ashamed to do the things that you were supposed to do … those things in front of his family. (Bongeka, 40-year-old woman, South Africa)

The strong influence of extended family on relationship dynamics was emphasized further by this participant: “Extended family, that's why we feel that it would have been beneficial to include that too, because we cannot run away from the fact that they have influences on the decisions, and how we make decisions” (Bongeka, 40-year-old woman, South Africa).

##### Joint chatbot use

Many participants also perceived that it would have been more beneficial if their partner also took part: “If we were in this together and then he would also have that ownership of ‘Okay, let's do this now, let's, you know.’ I really think it would have been best if he had taken, actually taken, part in it” (Vathiswa, 33-year-old woman, South Africa). While some participants preferred for both partners to take part, others, and particularly men, expressed a preference for partners to take part in the intervention separately: “I think it's good to do it on your own, but it would be beneficial to do it like, like separately, your partner does it on her phone and you do it on your phone” (Zolani, 27-year-old man, South Africa).

## Discussion

This is the first study, to our knowledge, that explores partnered parents’ perceptions and experiences of using a digital parenting intervention with integrated IPV prevention content. The study highlights the benefits of using a digital parenting program and the perceived shifts in relationship dynamics while recognizing some of the technological challenges of a chatbot. Three key themes were identified, which were related to the accessible and nonjudgmental perception of the chatbot, changes in relationship dynamics, and technological barriers and limitations.

The benefits associated with accessing parenting and relationship material via a chatbot were highlighted as a major advantage by participants. Interestingly, male participants emphasized how the digital modality of the chatbot allowed them to seek advice they would not have sought from any in-person sources. This is particularly notable given that male caregivers are often underrepresented in parenting interventions (Burn et al., 2019) and have, in past research, often been found to be difficult to involve in parenting programs (Stahlschmidt et al., 2013). The preference toward the digital format among the male caregivers in the present study is in line with existing research, which has found that internet-based and brief parenting programs were the most preferred delivery modalities among fathers (Frank et al., 2015; Tully et al., 2017). Indeed, flexibility and the option to schedule programs have been identified as key elements to encourage male caregiver engagement (Jensen et al., 2021). Research has also highlighted that barriers such as stigma and concerns about being judged are often associated with a lack of help-seeking (Tully et al., 2017). Given that stigma has been reported to be predictive of lower levels of help-seeking among low-income caregivers of children with behavioral problems (Dempster et al., 2015), flexible modalities, such as digital interventions, may offer an entry point to reach parents, including male caregivers, who might not otherwise seek support.

The relationship changes that participants perceived after using the chatbot, such as improved communication and reduced conflict, are also encouraging, and may be a reflection of the content of the chatbot. It is also possible that the content in the chatbot that emphasized adopting more equal distribution of household labor and more equitable gender norms around domestic roles could explain some of these subthemes that were reported in the findings. These changes are in line with indications of positive shifts in behaviors reported in other gender-transformative interventions, such as the Indashyikirwa program in Rwanda that works with couples, which found a reduction in conflict and improved trust and communication following an in-person intervention (Dunkle et al., 2020). The shared experiences of improvements in the division of household labor and decision-making in the present study are also in line with findings reported by other parenting interventions addressing VAW and VAC, such as the Bandebereho program in Rwanda (Doyle et al., 2023). Of note, however, the present study also revealed certain elements that were more resistant to change, such as perceptions around gender roles or equitable behaviors around extended family. These findings suggest that more deep-rooted beliefs surrounding gender roles, behaviors, and attitudes, may require additional content and engagement through, for example, a hybrid format (that is, a chatbot combined with facilitated, in-person sessions) or a community approach, to achieve long-term change.

Indeed, it may be that one-way engagement with a chatbot is insufficient to create changes in more deep-rooted behaviors and attitudes and that more two-way communication and interactive engagement are needed. One example of this approach includes group-based programming, which offers participants the opportunity to develop and apply the skills they learn in groups, and which may also help support greater levels of behavior change. For example, a systematic review of group-based parenting programs by Mathijs et al. (2024) found that in group settings, parents are able to learn from other parents, as well as provide and receive feedback, which may also improve self-confidence. As such, using a hybrid format, which combines digitally delivered content with in-person, group-based sessions, may therefore help shift more deep-rooted behaviors. Mathijs et al.'s (2024) review also suggests that certain mechanisms, such as social support and cohesion, may serve an important function in group-based interventions by, for example, helping parents feel a sense of connectedness. Notably, group cohesiveness may not only provide parents with the opportunity to learn from each other but might also help increase motivation and retention (Niec et al., 2005).

As such, while the digital format of ParentText offers various advantages over in-person programs, such as lower costs and greater reach, as discussed above, more deep-rooted norms and attitudes may require a more interactive, two-way, approach to achieve the intended behavior change. This need for greater levels of engagement to shift deep-rooted behaviors is underscored by results from a randomized trial of the digital intervention “ChattyCuz,” a relationship chatbot seeking to support young women in romantic relationships in South Africa (De Filippo et al., 2023). In the “ChattyCuz” study, it was found that only the gamified version of the chatbot led to significant, albeit modest, reductions in IPV, in comparison to the no-treatment group, which found no changes, and the narrative treatment group (that involved less engagement), which also had no effect on IPV (De Filippo et al., 2023).

Accordingly, a possible solution to addressing challenges with scale-up and delivery, while also achieving greater levels of engagement, might be to deliver digital parenting programs in virtual groups, rather than via in-person groups. An example of this approach is the Sharing Stories program, a digitally delivered parenting program facilitated via WhatsApp groups (Skeen et al., 2023). The program is based on a shared reading parenting intervention and the WHO's Thinking Healthy program, an evidence-based psychosocial intervention that seeks to improve mental health (Dowdall et al., 2021; Skeen et al., 2023; WHO, 2015). In the Sharing Stories program, caregivers of children aged 9–32 months, receive digital books through their phones and are provided with guidance on how to use the digital books to engage their children in playful ways (Skeen et al., 2023). The caregivers also receive content on strategies for coping with stress and for accessing support (Skeen et al., 2023). An RCT of Sharing Stories in Zambia and Tanzania found that, compared to the waitlist control, caregivers in the intervention group reported higher levels of responsive caregiving and significantly lower levels of depression and anxiety symptoms (Skeen et al., 2023). These findings suggest that digital parenting interventions delivered through WhatsApp-facilitated groups can help strengthen responsive caregiving as well as support caregiver mental health in low resource context, while also providing considerable potential for scalability.

Adopting a group-based format might also help with some of the technical issues faced in the present study. For example, various technological limitations of the chatbot were noted in the present research, which is in line with other existing studies on digital interventions. Indeed, while many participants expressed that the flexibility and acceptability associated with the digital modality was a major benefit of the chatbot, there were also limitations such as storage issues or lack of access to electricity that were seen as barriers. Notably, technical issues with digital interventions have been reported by other studies on digital interventions (Awah et al., 2022). As such, future studies should more closely examine how some of these technical and structural barriers can be addressed through, for example, hybrid approaches that combine digital and in-person modalities. Indeed, using a hybrid approach (that combines digital and in-person sessions), where facilitators are available to provide support, may improve program engagement and retention, since facilitators may be able to help resolve any issues that users encounter during the intervention, which may be harder to solve in a digital-only modality.

Taken together, these findings offer valuable insights into caregivers’ experiences of engaging with a digital chatbot, users’ perceptions of the structure of the chatbot, and changes that participants perceived in relationship dynamics after taking part in the program. In particular, the study sheds light on the promising potential of digital parenting interventions that contain IPV prevention content, while also highlighting areas that require further research.

### Limitations

This study also has limitations. The first relates to the selective sampling within the intervention and study. It is likely that caregivers choosing to take part in a parenting program are already more willing to change their behaviors than caregivers who may not have taken part. This sampling bias raises questions regarding how to reach caregivers who would benefit from the program, but who might be less willing to engage. The absence of male caregivers in the Jamaica sample also represents another limitation. More specifically, following challenges with male caregiver recruitment in Jamaica, only female caregivers took part in the intervention, with no male caregivers participating in Jamaica. Given research that underscores that it is critical to engage men and fathers in order to reduce IPV and VAC effectively, this is a notable limitation (Siu et al., 2017). Hence, going forward, adopting more targeted and male-specific recruitment strategies to ensure male caregivers and fathers participate is key (Gonzalez et al., 2023; McGirr et al., 2020; Panter-Brick et al., 2014). In the present study in Jamaica, participant recruitment was primarily conducted near schools, where mostly female caregivers would pick up and drop off children. As such, in the future, it would be important to include settings where more male caregivers are present (e.g., barbershops and sports centers), and use language-specific recruitment strategies, such as using the word “father” rather than “parent,” since research indicates that fathers frequently presume the word “parent” is interchangeable with “mother” (McGirr et al., 2020; Vollmer et al., 2019; Yaremych & Persky, 2023).

Moreover, various participants highlighted that they would have found the intervention to be more beneficial if their partner had also participated. In the future, it might therefore be worth exploring targeting couples together, rather than only targeting individual parents who are in relationships. One program that has adopted this approach is parenting for respectability (PfR), a group-based parenting program, that specifically recruits parental couples and fathers (Siu et al., 2024; Wight et al., 2022). Another notable approach adopted by PfR is the structure of the program, which in the first half consists of separate sessions for fathers and mothers, and in the second half consists of mixed-sex (couple-based) sessions. The initial single-sex session format provides participants with a safe space for both sexes to explore relationship topics that might be sensitive without fear of being judged and is a structure that especially fathers have expressed to be helpful (Siu et al., 2024). In addition to targeting couples, building on the present study's findings about the influence of relatives on relationship dynamics, future research would also benefit from exploring ways to engage extended family members. Given that restrictive beliefs of extended family were noted as a challenge when seeking to shift behaviors and attitudes, extending ParentText to relatives and other family members, might be helpful.

Another limitation is the setting of the interviews and FGDs in South Africa. While these were planned to be held in a meeting hall in a peri-urban township, due to safety concerns they were moved and held in a university meeting room. The university setting may have contributed to an increase in social desirability bias among participants, who may have not felt comfortable expressing less favorable experiences of ParentText while in an academic location. However, attempts to mitigate this were made by telling participants that there were no right or wrong answers and that any information shared was useful. In a similar vein, it is important to note the identity of the primary author, a Caucasian woman from a research institution in the Global North, who conducted the female interviews. While the interviews were carried out together with a local research assistant or member of staff, it is possible that participants may still not have felt comfortable sharing some of their experiences. Likewise, since some of the interviews were not conducted in English and were translated, it is possible some of the information shared was not captured. Efforts to address this were made by checking translations with the local research assistants and translators, however, it is possible certain nuances were not conveyed.

## Conclusion

The present research is the first qualitative study, to our knowledge, to examine caregivers’ experiences of engaging with a digital parenting intervention that includes integrated IPV prevention content. Findings from this study offer valuable insights into the potential of digital parenting interventions, with notable advantages identified including accessibility, anonymity, and perceived positive relationship changes. Technological limitations and barriers reported by participants raise important questions for consideration in future studies. Furthermore, the study highlights circumstances where a hybrid approach, which combines digitally delivered chatbot content with in-person, facilitated sessions, might be more beneficial (such as when seeking to shift more deep-rooted beliefs surrounding gender roles and equality), to achieve more long-term, sustainable change. Overall, the present study highlights the potential value of digitally delivered parenting programs in providing parents with greater flexibility surrounding how and when they engage with content, as well as in addressing barriers associated with attending in-person sessions, such as challenges with transport, scheduling difficulties, and time constraints. Notably, however, research evidence on the effectiveness of digital interventions in reducing IPV and VAC, and in reducing both forms of violence simultaneously, remains limited. As such, a key recommendation for future research includes conducting RCTs to test the causal effects of the intervention on both IPV and VAC, to compliment the qualitative findings from this study to better understand the effectiveness of the intervention on reducing IPV and VAC.

## Figures and Tables

**Figure 1.**
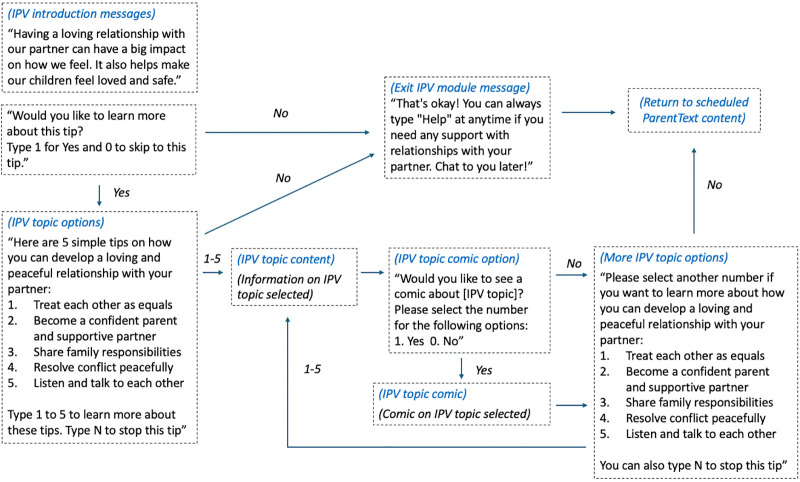
Flow of the IPV Prevention Content Text-Messages in ParentText

**Figure 2.**
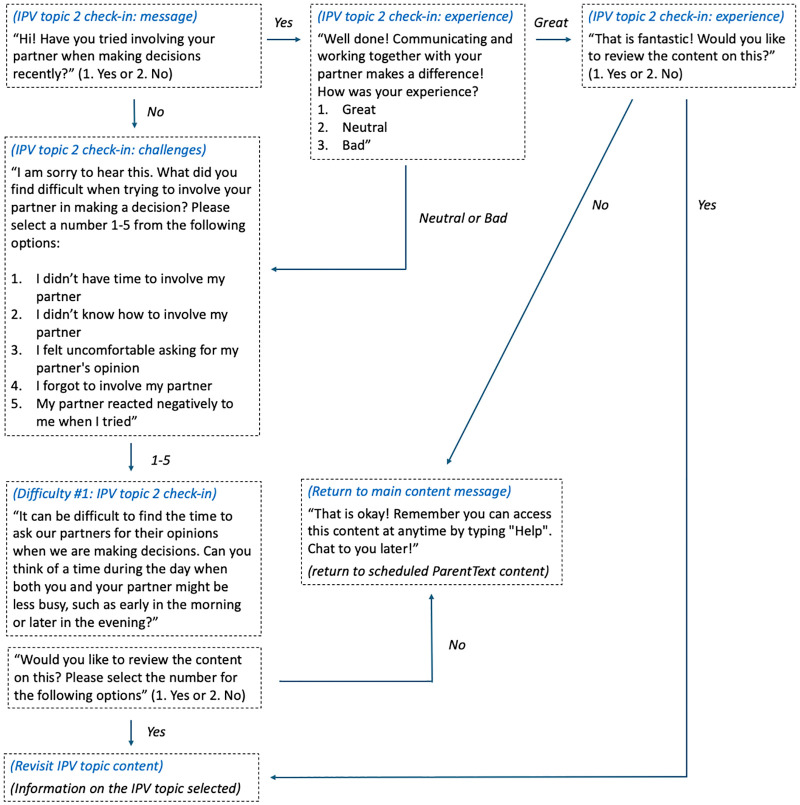
Flow of the Check-In Message on the IPV Topic #2 “Joint Decision-Making”

## Appendix A

Structure of the ParentText Intervention

**Table:**

Day	Morning	Main	Evening
1	**IPV Baseline Assessment**	Welcome	
2	**Content: IPV Main Material**	Supportive: Activities	
3	Supportive: Praise/Calm	Content: One on one time child	Supportive: Development
4	Supportive: Sharing	Content: Take a pause	**Check-in: IPV Topic 1 -** **Treat each other as equals**
5	Supportive: Praise/Calm	Content: Positive instructions	Supportive: Praise/Calm
6	Content: Take a pause	Supportive: Positive instructions	**Check-in: IPV Topic 2 -** **Become a confident parent and supportive spouse**
7	Check-in: Connection	Content: Praise	Supportive: Praise/Calm
8	Supportive: Praise/Calm	Content: Routines	**Check-in: IPV Topic 3 -** **Share family responsibilities**
9	Supportive: Praise/Calm	Supportive: Praise/Calm	Supportive: Praise/Calm
10	Supportive: Praise/Calm	Content: Positive rules	**Check-in: IPV Topic 4 -** **Resolve conflict peacefully**
11	Check-in: Covid	Content: Education	Supportive: Praise/Calm
12	Supportive: Praise/Calm	Content: Online	**Check-in: IPV Topic 5 -** **Listen and talk to each other**
13	Check-in: Rules	Content: Redirection	Supportive: Share
14	Supportive: Praise/Calm	Content: Behavior/crying	Content: Anger management 1
15	Check-in: Online	Content: Consequences	Check-in: One on one time
16	Supportive: Praise/Calm	Content: Ignore	Supportive: Praise/Calm
17	Supportive: Praise/Calm	Content: Emotion	Check-in: Praise
18	Check-in: Community safety	Supportive: Praise/Calm	Supportive: Praise/Calm
19	Supportive: Praise/Calm	Content: Budget with children	Supportive: Praise/Calm
20	Supportive: Praise/Calm	Supportive: Praise/Calm	Check-in: Positive
21	Check-in: Emotions	Content: Relax	Check-in: Ignore
22	Supportive: Praise/Calm	Content: Anger management 2	Supportive: Praise/Calm
23	Supportive: Praise/Calm	Check-in: Budget with children	Content: Safety
	Celebrate finishing program		

*Note.* This outline is the structure of the 23-day version of the ParentText Intervention. IPV prevention content is noted in bold font. Types of messages included in the program include content messages, check-in messages, and supportive messages.

## Appendix B

ParentText Intimate Partner Violence (IPV) Prevention Content

**Table:** A summary of the topics included in the IPV prevention content in ParentText is provided below. For each topic, there is also an example text message included. A full overview of the IPV prevention content in ParentText, including mother and gender-neutral versions of the material, is available in Schafer et al. (2023).

Topic	Content	Example message (fathers)
1	Treat each other as equals	“Family and friends might tell you how a husband or a father should act. But both men and women benefit when they talk to each other and make decisions together. For example, next time a decision needs to be made, involve your partner, and ask what they think!”
2	Become a confident parent and supportive spouse	“Get involved! When fathers are engaged in parenting their children, both the child, mother, and father benefit. Set aside some time today to spend with the children.”
3	Share family responsibilities	“Sharing family responsibilities with your partner can make life less stressful. Think of ways you can share the workload. Doing tasks together can also make them more fun.”
4	Resolve conflict peacefully	“All adults have disagreements sometimes. But fighting is not an effective way to solve issues. Instead, if you start feeling angry, take a deep breath first and then respond in a calmer way.”
5	Listen and talk to each other	“Listening and talking to those around us are key to a more peaceful home. Talking to your partner about issues before they become bigger problems can help avoid arguments from building up.”

## Appendix C

Overview of ParentText IPV Prevention Content

## Appendix D

FDG and Interview Question Guide

QUALITATIVE INSTRUMENT (ENGLISH):

[After introduction to the FDG or interview]: Thank you for participating in our program! It would help us to know more about your experience with the program. Each individual's unique experience is important to us. Any responses you give are very helpful. We would like to know about your experience during the program and how it has affected your family and relationships.

1. Could you tell me whether only you, or both you and your partner took part in the ParentText program?
  - What was your experience with this program?
  - If your partner also took part, did they tell you anything about their experience taking part in the program? Why do you think they participated?
2. Did you share what you were learning with anyone?
  - If yes Probe, Who? What did you share/talk about? Why did you share?
    – How about sharing with your partner?
    – How about sharing the content on relationship advice?
  - If no, why did you keep the learning to yourself?
3. Have you noticed any changes in your relationship with your partner following the program?
  - If yes, what changes have you noticed?
  - How about any changes in the way you interact with your partner?
  - How about in the way your partner interacts with you? Please tell me more…
4. Could you tell me a bit more about other parts of your relationship? Have you, for example, noticed any changes after the program in any of the following areas, such as:
  - In sharing the workload of household tasks or childcare with your partner?
  - In making decisions in the household, whether it be making decisions together with your partner or by yourself?
  - In experiencing or addressing disagreements or conflicts with your partner?
  - In talking or listening to your partner?
5. If no, why do you think there hasn’t been any changes in your relationship? Please tell me more…
6. Was there any specific part or content in the program that you liked or that you found helpful, especially in terms of your relationship with your partner? If so, can you tell me more about this?
7. Was there any specific part or content in the program that you did not like or that you found unhelpful, especially with regard to your relationship with your partner? If so, can you tell me more about this? Remember there are no right or wrong answers and any responses you give are very helpful.
8. Was there anything that you felt was missing from the program or that you would have liked to learn more about, particularly in terms of content about relationships?
9. Is there anything else you would like to share about your experience on using ParentText and any potential impact it did or did not have on your relationship with your partner?

Thank you so much again for taking the time to participate in the program and for taking the time to answer these questions. We really appreciate it!

## References

[bibr1-10778012251329363] Alhusen J. L. Ho G. W. K. Smith K. F. Campbell J. C. (2014). Addressing intimate partner violence and child maltreatment: Challenges and opportunities. In Korbin J. E. Krugman R. D. (Eds.), Handbook of child maltreatment (pp. 327–349). Springer.

[bibr2-10778012251329363] Ambrosio M. D. G. Lachman J. M. Zinzer P. Gwebu H. Vyas S. Vallance I. Calderon F. Gardner F. Markle L. Stern D. Facciola C. Schley A. Danisa N. Brukwe K. Melendez-Torres G. J. (2024). A factorial randomized controlled trial to optimize user engagement with a chatbot-led parenting intervention: Protocol for the parentText optimisation trial. JMIR Research Protocols, 13(1), e52145. 10.2196/52145 PMC11102037 38700935

[bibr3-10778012251329363] Anderson E. J. McClelland J. Meyer Krause C. Krause K. C. Garcia D. O. Koss M. P. (2019). Web-based and mHealth interventions for intimate partner violence prevention: A systematic review protocol. BMJ Open, 9(8), 1–5. 10.1136/bmjopen-2019-029880 PMC6701589 31401604

[bibr4-10778012251329363] Awah I. Green O. Baerecke L. Janowski R. Klapwijk J. Chetty A. N. Wamoyi J. Cluver L. D. (2022). ‘It provides practical tips, practical solutions!’: Acceptability, usability, and satisfaction of a digital parenting intervention across African countries. Psychology, Health & Medicine, 27(sup1), 107–123. 10.1080/13548506.2022.2113106 35980251

[bibr5-10778012251329363] Backhaus S. Gardner F. Schafer M. Melendez-Torres G. J. Knerr W. Lachman J. M. (2023). Parenting interventions to prevent child maltreatment and enhance parent-child relationships with children aged 0-17 years. Report of the systematic reviews of evidence. https://cdn.who.int/media/docs/default-source/documents/violence-prevention/systematic_reviews-for-the-who-parenting-guideline-jan-27th-2023.pdf?sfvrsn=158fd424_3

[bibr6-10778012251329363] Braun V. Clarke V. (2006). Using thematic analysis in psychology. Qualitative Research in Psychology, 3(2), 77–101. 10.1191/1478088706qp063oa

[bibr7-10778012251329363] Braun V. Clarke V. (2021). Conceptual and design thinking for thematic analysis. Qualitative Psychology, 9(1), 3–26. 10.1037/qup0000196

[bibr8-10778012251329363] Burke S. (2021). Rethinking ‘validity’ and ‘trustworthiness’ in qualitative inquiry: How might we judge the quality of qualitative research in sport and exercise sciences? In Routledge handbook of qualitative research in sport and exercise (pp. 352–362). Taylor & Francis. 10.4324/9781315762012-37

[bibr9-10778012251329363] Burn M. Tully L. A. Jiang Y. Piotrowska P. J. Collins D. A. J. Sargeant K. Hawes D. Moul C. Lenroot R. K. Frick P. J. Anderson V. Kimonis E. R. Dadds M. R. (2019). Evaluating practitioner training to improve competencies and organizational practices for engaging fathers in parenting interventions. Child Psychiatry and Human Development, 50(2), 230–244. 10.1007/S10578-018-0836-2/TABLES/3 30078112 PMC6428790

[bibr10-10778012251329363] Casey E. Carlson J. Two Bulls S. Yager A. (2018). Gender transformative approaches to engaging men in gender-based violence prevention: A review and conceptual model. Trauma, Violence, & Abuse, 19(2), 231–246. 10.1177/1524838016650191 27197533

[bibr11-10778012251329363] Cluver L. D. Meinck F. Steinert J. I. Shenderovich Y. Doubt J. Romero R. H. Lombard C. J. Redfern A. Ward C. L. Tsoanyane S. Nzima D. Sibanda N. Wittesaele C. De Stone S. Boyes M. E. Catanho R. Lachman J. M. L. Salah N. Nocuza M. Gardner F. (2018). Parenting for lifelong health: A pragmatic cluster randomised controlled trial of a non-commercialised parenting programme for adolescents and their families in South Africa. BMJ Global Health, 3(1), 1–15. 10.1136/bmjgh-2017-000539 PMC5859808 29564157

[bibr12-10778012251329363] Cyr J. (2015). The pitfalls and promise of focus groups as a data collection method. Sociological Methods & Research, 45(2), 231–259. 10.1177/0049124115570065

[bibr13-10778012251329363] David O. A. Iuga I. A. Miron I. S. (2024). Parenting: There is an app for that. A systematic review of parenting interventions apps. Children and Youth Services Review, 156, 107385. 10.1016/J.CHILDYOUTH.2023.107385

[bibr14-10778012251329363] De Filippo A. Bellatin P. Tietz N. Grant E. Whitefield A. Nkopane P. Devereux C. Crawford K. Vermeulen B. Hatcherid A. M. (2023). Effects of digital chatbot on gender attitudes and exposure to intimate partner violence among young women in South Africa. PLOS Digital Health, 2(10), e0000358. 10.1371/JOURNAL.PDIG.0000358 PMC10578594 37844088

[bibr15-10778012251329363] Dempster R. Davis D. W. Faye Jones V. Keating A. Wildman B. (2015). The role of stigma in parental help-seeking for perceived child behavior problems in urban, low- income African American parents. Journal of Clinical Psychology in Medical Settings, 22(4), 265–278. 10.1007/S10880-015-9433-8 26370202

[bibr16-10778012251329363] Dowdall N. Murray L. Skeen S. Marlow M. De Pascalis L. Gardner F. Tomlinson M. Cooper P. J. (2021). Book-Sharing for parenting and child development in South Africa: A randomized controlled trial. Child Development, 92(6), 2252–2267. 10.1111/CDEV.13619 34716710

[bibr17-10778012251329363] Doyle K. Levtov R. G. Barker G. Bastian G. G. Bingenheimer J. B. Kazimbaya S. Nzabonimpa A. Pulerwitz J. Sayinzoga F. Sharma V. Shattuck D. (2018). Gender- transformative bandebereho couples’ intervention to promote male engagement in reproductive and maternal health and violence prevention in Rwanda: Findings from a randomized controlled trial. PLoS ONE, 13(4), 1–17. 10.1371/journal.pone.0192756 PMC5884496 29617375

[bibr18-10778012251329363] Doyle K. Levtov R. G. Karamage E. Rakshit D. Kazimbaya S. Sayinzoga F. Sibomana H. Ngayaboshya S. Rutayisire F. Barker G. (2023). Long-term impacts of the Bandebereho programme on violence against women and children, maternal health-seeking, and couple relations in Rwanda: A six-year follow-up of a randomised controlled trial. EClinicalMedicine, 64, 102233. 10.1016/j.eclinm.2023.102233 37781160 PMC10539919

[bibr19-10778012251329363] Dunkle K. Stern E. Chatterji S. Heise L. (2020). Effective prevention of intimate partner violence through couples training: A randomised controlled trial of Indashyikirwa in Rwanda. BMJ Global Health, 5(12). 10.1136/bmjgh-2020-002439 PMC7757483 33355268

[bibr20-10778012251329363] Frank T. J. Keown L. J. Dittman C. K. Sanders M. R. (2015). Using father preference data to increase father engagement in evidence-based parenting programs. Journal of Child and Family Studies, 24(4), 937–947. 10.1007/s10826-014-9904-9

[bibr21-10778012251329363] Gibbs A. Washington L. Abdelatif N. Chirwa E. Willan S. Shai N. Sikweyiya Y. Mkhwanazi S. Ntini N. Jewkes R. (2020). Stepping stones and creating futures intervention to prevent intimate partner violence among young people: Cluster randomized controlled trial. Journal of Adolescent Health, 66(3), 323–335. 10.1016/J.JADOHEALTH.2019.10.004 31784410

[bibr22-10778012251329363] Given L. (2008). The SAGE Encyclopedia of Qualitative Research Methods. Sage Publications, Inc.. 10.4135/9781412963909

[bibr23-10778012251329363] Gonzalez J. C. Klein C. C. Barnett M. L. Schatz N. K. Garoosi T. Chacko A. Fabiano G. A. (2023). Intervention and implementation characteristics to enhance father engagement: A systematic review of parenting interventions. Clinical Child and Family Psychology Review, 26(2), 445–458. 10.1007/s10567-023-00430-x 36947287 PMC10031187

[bibr24-10778012251329363] Gordon C. (2016). Intimate partner violence is everyone’s problem, but how should we approach it in a clinical setting? South African Medical Journal, 106(10), 962–965. 10.7196/SAMJ.2016.V106I10.11408

[bibr25-10778012251329363] Gracia E. Rodriguez C. M. Martín-Fernández M. Lila M. (2017). Acceptability of family violence: Underlying ties between intimate partner violence and child abuse. Journal of Interpersonal Violence, 35(17-18), 3217–3236. 10.1177/0886260517707310 29294751

[bibr26-10778012251329363] Guedes A. Bott S. Garcia-Moreno C. Colombini M. (2016). Bridging the gaps: A global review of intersections of violence against women and violence against children. Global Health Action, 9(1), 31516. 10.3402/gha.v9.31516 27329936 PMC4916258

[bibr27-10778012251329363] Hall A. K. Cole-Lewis H. Bernhardt J. M. (2015). Mobile text messaging for health: A systematic review of reviews. Annual Review of Public Health, 36(1), 393–415. 10.1146/annurev-publhealth-031914-122855 PMC4406229 25785892

[bibr28-10778012251329363] Hillis S. D. Mercy J. A. Saul J. R. (2017). The enduring impact of violence against children. Psychology, Health and Medicine, 22(4), 393–405. 10.1080/13548506.2016.1153679 26979496

[bibr29-10778012251329363] Jensen S. K. Placencio-Castro M. Murray S. M. Brennan R. T. Goshev S. Farrar J. Yousafzai A. Rawlings L. B. Wilson B. Habyarimana E. Sezibera V. Betancourt T. S. (2021). Effect of a home-visiting parenting program to promote early childhood development and prevent violence: A cluster-randomized trial in Rwanda. BMJ Global Health, 6(1), e003508. 10.1136/BMJGH-2020-003508 PMC7849888 33514591

[bibr30-10778012251329363] Korstjens I. Moser A. (2018). Series: Practical guidance to qualitative research. Part 4: Trustworthiness and publishing. European Journal of General Practice, 24(1), 120–124. 10.1080/13814788.2017.1375092 29202616 PMC8816392

[bibr31-10778012251329363] Kruger L. J. Rodgers R. F. Long S. J. Lowy A. S. (2019). Individual interviews or focus groups? Interview format and women’s self-disclosure. International Journal of Social Research Methodology, 22(3), 245–255. 10.1080/13645579.2018.1518857

[bibr32-10778012251329363] Lambert S. D. Loiselle C. G. (2008). Combining individual interviews and focus groups to enhance data richness. Journal of Advanced Nursing, 62(2), 228–237. 10.1111/J.1365-2648.2007.04559.X 18394035

[bibr33-10778012251329363] Levy J. K. Darmstadt G. L. Ashby C. Quandt M. Halsey E. Nagar A. Greene M. E. (2020). Characteristics of successful programmes targeting gender inequality and restrictive gender norms for the health and wellbeing of children, adolescents, and young adults: A systematic review. The Lancet Global Health, 8(2), e225–e236. 10.1016/S2214-109X(19)30495-4 PMC7025324 31879212

[bibr34-10778012251329363] Lincoln Y. S. Guba E. G. (1985). Naturalistic inquiry. Sage.

[bibr35-10778012251329363] Marcos-Marcos J. Nardini K. Briones-Vozmediano E. Vives-Cases C. (2023). Listening to stakeholders in the prevention of gender-based violence among young people in Spain: A qualitative study from the positivMasc project. BMC Women’s Health, 23(1), 1–13. 10.1186/S12905-023-02545-3 37496067 PMC10373224

[bibr36-10778012251329363] Mathijs L. Van Petegem S. Melendez-Torres G. J. Backhaus S. Gardner F. Leijten P. (2024). Group-Based versus individual parenting programs: A meta-analysis of effects on parents. Journal of Family Psychology, 38(8), 1109–1118. 10.1037/fam0001273 39432337

[bibr37-10778012251329363] McGirr S. Torres J. Heany J. Brandon H. Tarry C. Robinson C. (2020). Lessons learned on recruiting and retaining young fathers in a parenting and repeat pregnancy prevention program. Maternal and Child Health Journal, 24(2), 183–190. 10.1007/s10995-020-02956-w 32564249 PMC7497383

[bibr38-10778012251329363] Niec L. N. Hemme J. M. Yopp J. M. Brestan E. V. (2005). Parent-child interaction therapy: The rewards and challenges of a group format. Cognitive and Behavioral Practice, 12(1), 113–125. 10.1016/S1077-7229(05)80046-X

[bibr39-10778012251329363] OECD . (2023). *Violence against women (indicator)*. 10.1787/f1eb4876-en

[bibr40-10778012251329363] Opie J. E. Esler T. B. Clancy E. M. Wright B. Painter F. Vuong A. Booth A. T. Newman L. Johns-Hayden A. Hameed M. Hooker L. Olsson C. McIntosh J. E. (2023). Universal digital programs for promoting mental and relational health for parents of young children: A systematic review and meta-analysis. Clinical Child and Family Psychology Review, 27(1), 23–52. 10.1007/S10567-023-00457-0 37917315 PMC10920439

[bibr41-10778012251329363] Oram S. Fisher H. L. Minnis H. Seedat S. Walby S. Hegarty K. Rouf K. Angénieux C. Callard F. Chandra P. S. Fazel S. Garcia-Moreno C. Henderson M. Howarth E. MacMillan H. L. Murray L. K. Othman S. Robotham D. Rondon M. B. Howard L. M. (2022). The lancet psychiatry commission on intimate partner violence and mental health: Advancing mental health services, research, and policy. The Lancet Psychiatry, 9(6), 487–524. 10.1016/S2215-0366(22)00008-6/35569504

[bibr42-10778012251329363] Panter-Brick C. Burgess A. Eggerman M. McAllister F. Pruett K. Leckman J. F. (2014). Practitioner review: Engaging fathers – recommendations for a game change in parenting interventions based on a systematic review of the global evidence. Journal of Child Psychology and Psychiatry, and Allied Disciplines, 55(11), 1187. 10.1111/JCPP.12280 24980187 PMC4277854

[bibr43-10778012251329363] Pearson I. Page S. Zimmerman C. Meinck F. Gennari F. Guedes A. Stöckl H. (2022). The Co-Occurrence of Intimate Partner Violence and Violence Against Children: A Systematic Review on Associated Factors in Low- and Middle-Income Countries. Trauma, Violence, and Abuse, 24(4). 10.1177/15248380221082943 PMC10486154 35481390

[bibr44-10778012251329363] Prevention Collaborative. (2023). Pathways between poor mental health and intimate partner violence (Issue February). https://prevention-collaborative.org/wp-content/uploads/2023/02/4_Mental-Health-Evidence-Review-2024-update.pdf Prevention Collaborative.

[bibr45-10778012251329363] QRS International . (2023). *NVivo Version 14*. www.lumivero.com

[bibr46-10778012251329363] Rohleder P. Lyons A. (2017). Qualitative research in clinical and health psychology. Bloomsbury Publishing.

[bibr47-10778012251329363] Sanders M. R. Divan G. Singhal M. Turner K. M. T. Velleman R. Michelson D. Patel V. (2021). Scaling up parenting interventions is critical for attaining the sustainable development goals. Child Psychiatry and Human Development, 53(5), 1–12. 10.1007/s10578-021-01171-0 PMC8096135 33948778

[bibr48-10778012251329363] Schafer M. Lachman J. M. Gardner F. Zinser P. Calderon F. Han Q. Facciola C. Clements L. (2023). Integrating intimate partner violence prevention content into a digital parenting chatbot intervention during COVID-19: Intervention development and remote data collection. BMC Public Health, 23(1), 1–17. 10.1186/S12889-023-16649-W 37667352 PMC10476288

[bibr49-10778012251329363] Schafer M. Lachman J. Zinser P. Calderón Alfaro F. A. Han Q. Facciola C. Clements L. Gardner F. Haupt Ronnie G. Sheil R. (2025). A digital parenting intervention with intimate partner violence prevention content: Quantitative Pre-post pilot study. JMIR Formative Research, 9(1), e58611. 10.2196/5861 PMC11748420 39753219

[bibr50-10778012251329363] Sere Y. Roman N. V. Ruiter R. A. C. (2021). Coping with the experiences of intimate partner violence among South African women: Systematic review and meta-synthesis. Frontiers in Psychiatry, 12, 522. 10.3389/FPSYT.2021.655130/BIBTEX PMC8187566 34122178

[bibr51-10778012251329363] Shai N. J. Sikweyiya Y. (2015). Programmes for change - addressing sexual and intimate partner violence in South Africa. SA Crime Quarterly, 51, 31–41. 10.4314/sacq.v51i1.4

[bibr52-10778012251329363] Siu G. Nsubuga R. N. Lachman J. M. Namutebi C. Sekiwunga R. Zalwango F. Riddell J. Wight D. (2024). The impact of the parenting for respectability programme on violent parenting and intimate partner relationships in Uganda: A pre-post study. PLoS ONE, 19(5 May), 1–16. 10.1371/journal.pone.0299927 PMC11125497 38787892

[bibr53-10778012251329363] Siu G. E. Wight D. Seeley J. Namutebi C. Sekiwunga R. Zalwango F. Kasule S. (2017). Men’s involvement in a parenting programme to reduce child maltreatment and gender-based violence: Formative evaluation in Uganda. European Journal of Development Research, 29(5), 1017–1037. 10.1057/s41287-017-0103-6

[bibr54-10778012251329363] Skeen S. Marlow M. du Toit S. Melendez-Torres G. J. Mudekunye L. Mapalala E. Ngoma K. Ntanda B. M. Maketha M. Grieve C. Hartmann L. Gordon S. Tomlinson M. (2023). Using WhatsApp support groups to promote responsive caregiving, caregiver mental health and child development in the COVID-19 era: A randomised controlled trial of a fully digital parenting intervention. Digital Health, 9, 10.1177/20552076231203893 PMC10624105 37928327

[bibr55-10778012251329363] Skivington K. Matthews L. Simpson S. A. Craig P. Baird J. Blazeby J. M. Boyd K. A. Craig N. French D. P. McIntosh E. Petticrew M. Rycroft-Malone J. White M. Moore L. (2021). A new framework for developing and evaluating complex interventions: update of Medical Research Council guidance. BMJ, 374, 10.1136/BMJ.N2061 PMC8482308 34593508

[bibr56-10778012251329363] Stahlschmidt M. J. Threlfall J. Seay K. D. Lewis E. M. Kohl P. L. (2013). Recruiting fathers to parenting programs: Advice from dads and fatherhood program providers. Children and Youth Services Review, 35(10), 1734–1741. 10.1016/j.childyouth.2013.07.004 24791035 PMC4002003

[bibr57-10778012251329363] Trevillion K. Oram S. Feder G. Howard L. M. (2012). Experiences of domestic violence and mental disorders: A systematic review and meta-analysis. PLoS ONE, 7(12), e51740. 10.1371/JOURNAL.PONE.0051740 PMC3530507 23300562

[bibr58-10778012251329363] Tully L. A. Piotrowska P. J. Collins D. A. J. Mairet K. S. Black N. Kimonis E. R. Hawes D. J. Moul C. Lenroot R. K. Frick P. J. Anderson V. Dadds M. R. (2017). Optimising child outcomes from parenting interventions: Fathers’ experiences, preferences and barriers to participation. BMC Public Health, 17(1), 1–14. 10.1186/S12889-017-4426-1 28592244 PMC5463495

[bibr59-10778012251329363] UNICEF. (2020). COVID-19 Risk Communication and Community Engagement (RCCE) Guidance Note on Quantitative Data Collection. www.corecommitments.unicef.org/kp/rcce-covid19—quantitative-data-collection- guidance-note.pdf

[bibr60-10778012251329363] UNICEF Innocenti, Prevention Collaborative, & Equimundo. (2023). Parenting Programmes to Reduce Violence against Children and Women. Why it is important. Brief 1. UNICEF Innocenti. https://www.unicef-irc.org/publications/pdf/UNICEF-Innocenti- Parenting-Brief-1-Why-is-it-important-2024.pdf

[bibr61-10778012251329363] Vollmer R. L. Adamsons K. Mobley A. R. (2019). Recruitment, engagement, and retention of fathers in nutrition education and obesity research. Journal of Nutrition Education and Behavior, 51(9), 1121–1125. 10.1016/j.jneb.2019.07.006 31378688 PMC6788966

[bibr62-10778012251329363] Wang R. Zhu B. Yu X. Tan W. Shi Q. (2025). Childhood violence exposure and anxiety and depression of children and adolescents. Journal of Affective Disorders, 369, 608–614. 10.1016/J.JAD.2024.10.044 39406297

[bibr63-10778012251329363] Ward C. L. Wessels I. M. Lachman J. M. Hutchings J. Cluver L. D. Kassanjee R. Nhapi R. Little F. Gardner F. (2020). Parenting for lifelong health for young children: A randomized controlled trial of a parenting program in South Africa to prevent harsh parenting and child conduct problems. Journal of Child Psychology and Psychiatry, 61(4), 503–512. 10.1111/jcpp.13129 31535371 PMC7155004

[bibr64-10778012251329363] WHO. (2015). Thinking healthy: A manual for psychosocial management of perinatal depression. World Health Organization, Vol. 1. https://iris.who.int/bitstream/handle/10665/152936/?sequence=1

[bibr65-10778012251329363] WHO. (2021). *Violence against women prevalence estimates, 2018:* global, regional and national prevalence estimates for intimate partner violence against women and global and regional prevalence estimates for non-partner sexual violence against women. https://iris.who.int/bitstream/handle/10665/341338/9789240026681-eng.pdf

[bibr66-10778012251329363] WHO . (2022). *WHO guidelines on parenting interventions to prevent maltreatment and enhance parent–child relationships with children aged 0–17 years*. https://www.who.int/publications/i/item/9789240065505

[bibr67-10778012251329363] Wight D. Sekiwunga R. Namutebi C. Zalwango F. Siu G. E. (2022). A Ugandan parenting programme to prevent gender-based violence: Description and formative evaluation. Research on Social Work Practice, 32(4), 448–464. 10.1177/10497315211056246 35431527 PMC7612614

[bibr68-10778012251329363] Yaremych H. E. Persky S. (2023). Recruiting fathers for parenting research: An evaluation of eight recruitment methods and an exploration of Fathers’ motivations for participation. Parenting, 23(1), 1–32. 10.1080/15295192.2022.2036940 37346458 PMC10281717

